# Microbiome-behavior coupling shapes infant adaptation to early maternal unpredictability

**DOI:** 10.3389/fmicb.2026.1830339

**Published:** 2026-06-02

**Authors:** Dima Amso, Guilherme Fahur Bottino, Tess T. Forest, Kevin S. Bonham, Michal R. Zieff, Fadheela Patel, Marlie Miles, Donna Herr, Khula South Africa Data Collection Team, Claudia Espinoza-Heredia, Celia L. D’Amato, Jinge Ren, Melissa Gladstone, Emmie Mbale, Daniel C. Alexander, Derek K. Jones, Steve C. R. Williams, William P. Fifer, Laurel J. Gabard-Durnam, Kirsten A. Donald, Vanja Klepac-Ceraj

**Affiliations:** 1 Department of Psychology, Columbia University, New York, NY, United States; 2 Department of Biological Sciences, Wellesley College, Wellesley, MA, United States; 3 Department of Medicine, Tufts Medical Center, Boston, MA, United States; 4 University of Cape Town, Cape Town, Western Cape, South Africa; 5 SAMRC/CPUT/Cardiometabolic Health Research Unit, Department of Biomedical Sciences, Faculty of Health and Wellness Sciences, Cape Peninsula University of Technology, Bellville, South Africa; 6 Department of Women and Children’s Health, Institute of Life Course and Medical Science, Alder Hey Children’s NHS Foundation Trust, University of Liverpool, Liverpool, United Kingdom; 7 Department of Paediatrics and Child Health, Kamuzu University of Health Science, Blantyre, Malawi; 8 UCL Hawkes Institute and Department of Computer Science, University College London, London, United Kingdom; 9 Cardiff University Brain Research Imaging Centre, Cardiff University, Cardiff, Wales, United Kingdom; 10 Department of Neuroimaging, King’s College London, London, United Kingdom; 11 Department of Psychiatry, Columbia University Irving Medical Center, NYSPI, New York, NY, United States; 12 Department of Psychology, Northeastern University, Boston, MA, United States; 13 Department of Pediatrics and Child Health, Red Cross War Memorial Children’s Hospital, Cape Town, Western Cape, South Africa; 14 Neuroscience Institute, University of Cape Town, Cape Town, Western Cape, South Africa

**Keywords:** behavior, *Bifidobacterium breve*, *Bifidobacterium longum*, caretaker-child interaction, infant gut microbiome, maternal unpredictability, phenotypic plasticity, visual orienting behavior

## Abstract

How do some human infants adapt to environmental challenges while others do not? We examined whether infant behavioral responses to maternal unpredictability predict early inhibitory control and are linked to gut microbial community composition and neuroactive metabolic potential. Maternal unpredictability, quantified as the entropy of sensory signal transitions during mother-infant interaction (*N* = 255; 2–6 months), predicted poorer infant inhibitory control at 19–28 months. However, infants who exhibited high visual orienting behavior (VOB) under high unpredictability showed later inhibitory control comparable to infants exposed to low unpredictability, suggesting an adaptive behavioral buffering strategy. In a subset of infants (*n* = 87), we tested whether infant age, sex, delivery mode, feeding, maternal education, and maternal unpredictability explained variation in gut microbial community diversity. Only feeding status and VOB were significantly associated with both taxonomic and functional microbial profiles. VOB was associated with taxonomic and functional variation along a *Bifidobacterium breve* and *Bifidobacterium longum* axis and enrichment of microbial tryptophan and glutamate synthesis genes. Although feeding groups differed in alpha diversity, VOB was not associated with feeding status, suggesting that feeding is not the primary driver of the observed VOB-microbiome signatures. Interaction models of neuroactive gene functions revealed that microbial signatures vary across combinations of VOB and maternal unpredictability, suggesting that the microbial support for deploying visual attentional strategies differs under distinct levels of environmental unpredictability. Together, these findings support a framework in which infant behavioral strategy is associated with variation in gut microbial composition and metabolic gene potential.

## 1 Introduction

Early childhood experiences shape later cognitive and mental health outcomes (Shonkoff and Phillips, 2000; Knudsen, 2004). However, the biological mechanisms linking early life-exposures to developmental variability in humans remain poorly understood. These influences are almost always probabilistic rather than deterministic (Gottlieb, 1991), pointing to the importance of understanding the sources of individual variability in developmental trajectories. Phenotypic plasticity, the capacity of a single genotype to produce different phenotypes, provides a framework for understanding such variation in human cognition and development (Pigliucci, 2001). Across species, environmental unpredictability shapes adaptive behavioral traits, from foraging and aggression (Marcotte and Browman, 1986; Ab Ghani and Merilä, 2015; Herczeg et al., 2016; Schradin et al., 2019) to altered stress physiology (Ellis et al., 2009; Noroña-Zhou et al., 2020), to learning and executive control (Davis et al., 2017; Forest et al., 2025b), suggesting that variability in early conditions can canalize different developmental solutions (Frankenhuis and de Weerth, 2013; Botero et al., 2015). Here we investigated the associations between maternal unpredictability and human infant gut microbial composition and behavioral responses, and whether these associations are consistent with candidate mechanisms of phenotypic plasticity in early human development.

Humans are altricial, meaning that infants rely on caregivers for survival and learning (Tottenham, 2015). A substantial literature shows that caregivers differ in the predictability of behavioral sequences delivered to infants during daily interaction, and that this behavioral unpredictability is measurable from temporal transitions among caregiver sensory behaviors (Davis et al., 2017). High maternal unpredictability has been associated with poorer later learning, self-regulation, and inhibitory control in both animal models and human work (Davis et al., 2017; Glynn and Baram, 2019; Goodwill et al., 2019; Manzano Nieves et al., 2019; Holmberg et al., 2022; Aran et al., 2024). In previous work in the cohort used in this analysis (Zieff et al., 2024), infants tested in two separate countries (Malawi, South Africa) dynamically adjusted their looking times based on moment-to-moment changes in their caregiver’s unpredictability (Forest et al., 2025a), in a manner that is consistent with online optimization of attention in the service of learning (Kidd et al., 2012). Indeed, in the same cohort, caregiver unpredictability in very early infancy was associated with reduced neural signatures of infants’ statistical learning months later (Forest et al., 2025b). Thus, precisely how infants behaviorally respond to early life maternal unpredictability *in the moment,* and the mechanisms that enable this response, may help clarify how maternal unpredictability relates to later developmental outcomes.

Thus, we focused this analysis on infants’ moment-to-moment behavior, operationalized as visual orienting behavior (VOB) shifts to and from the mother during naturalistic dyadic interactions. This choice is grounded in classic findings that infants rapidly extract regularities and adjust visual attention orienting behavior to statistical structure in their environment (Saffran et al., 1996; Kirkham et al., 2002; Aslin, 2017). Why might this impact later self-regulation and inhibitory control? Development proceeds along broad caudal to rostral cortical gradients: early-maturing sensory cortices such as vision can influence, and be integrated with, later-developing control systems, including the prefrontal cortex (Gilbert and Li, 2013; Amso and Scerif, 2015; Johnson et al., 2015). Thus, we expected that differences in early VOB response to the mother, enabling or precluding learning in the moment, could be one pathway by which early experience with maternal unpredictability is associated with later developing inhibitory control outcomes, though causal inference is beyond the scope of the present study. Building on that rationale, we assessed whether infants exposed to more unpredictable maternal sequences adjust their VOB patterns in ways that forecast later inhibitory control skills (Davis et al., 2017; Glynn and Baram, 2019; Aran et al., 2024). A related question relates to understanding whether the gut microbial community plays a role in these behavioral responses.

Although several studies have independently linked infant behavior to later developmental outcomes, whether infant behavioral responses to early environmental unpredictability are associated with gut microbial composition remains largely unexplored. The infant gut microbiome is dynamic in the first months of life and typically dominated by *Bifidobacterium* species whose relative abundance varies across infants, with recurrent patterns across geographies (Dominguez-Bello et al., 2010; Bäckhed et al., 2015; Fahur Bottino et al., 2025). Microbial metabolites, including short-chain fatty acids (SCFAs), glutamate/GABA, and tryptophan-derived indoles/kynurenines, have been implicated in modulation of microglia, excitation/inhibition (E/I) balance, and sensitive-period plasticity (Cryan and Dinan, 2012; Erny et al., 2015; Janik et al., 2016; Cryan et al., 2019; Valles-Colomer et al., 2019; Ahmed et al., 2022). These gut-brain signaling pathways, active during a period of rapid cortical development, may thus represent one mechanism through which infant behavioral responses to environmental predictability are implemented and sustained.

**Figure 1:**
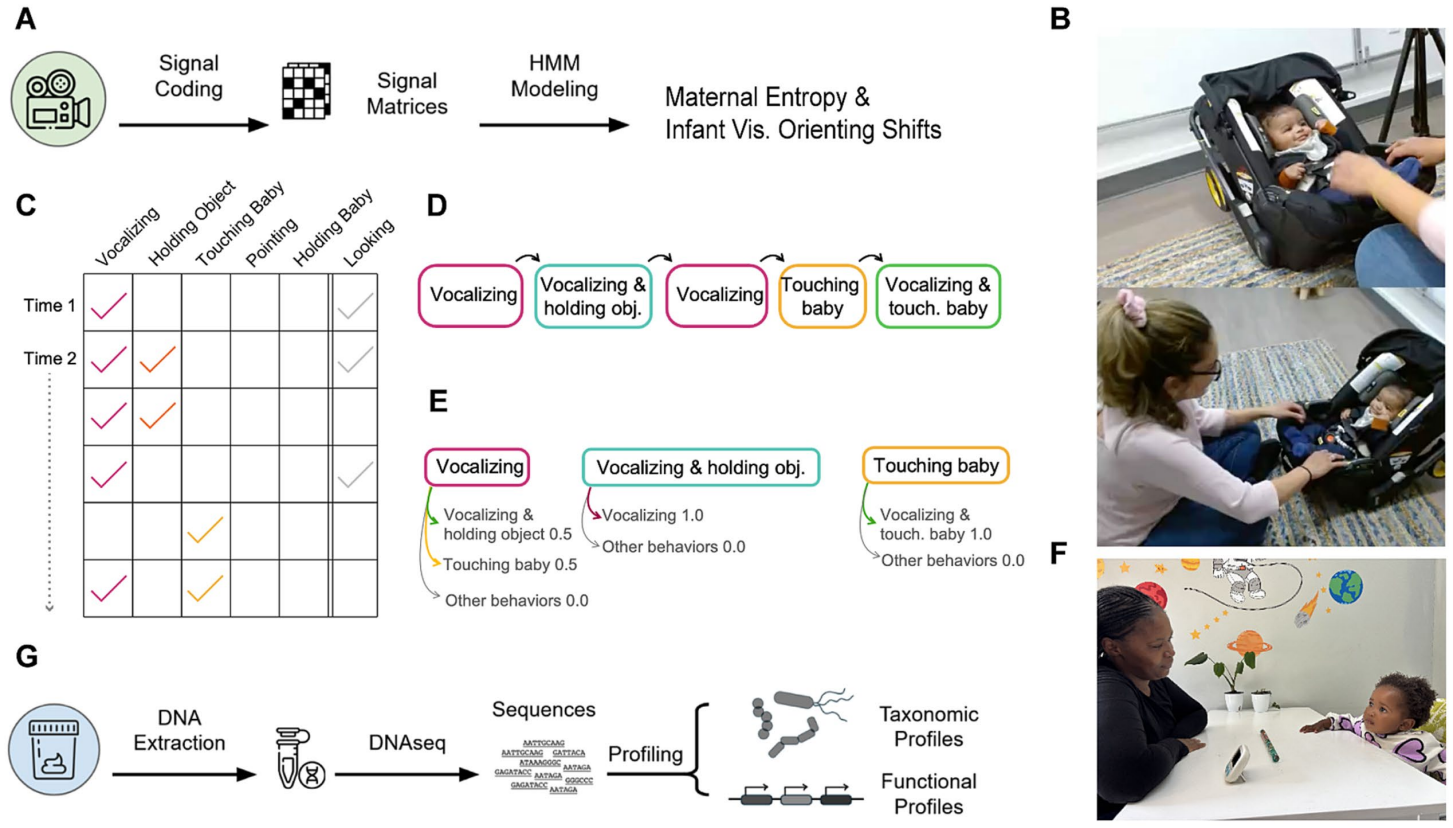
Study design integrating mother-infant interaction dynamics, entropy modeling, gut microbiome profiling, and later executive function assessment. **(A)** Video recordings of mother-infant dyadic interactions were obtained when infants were between 2 and 6 months of age to quantify infant visual orienting behavior (VOB) and maternal behavioral entropy (unpredictability). **(B)** Representative images of caregiver-infant interactions during recording. **(C)** Videos were hand-coded frame-by-frame for maternal behaviors (e.g., vocalizing, holding objects, touching the baby) and concurrent infant visual orienting shifts. **(D)** Coded behaviors were transformed into discrete behavioral states and ordered as temporal sequences. **(E)** Caregiver unpredictability (Shannon entropy) was computed from behavioral state transition probabilities derived from these sequences. **(F)** Infant stool samples were collected within one month of the recorded interaction and underwent DNA extraction and sequencing, generating taxonomic and functional microbiome profiles. **(G)** At 19–28 months of age, children completed an inhibitory control task (glitter wand) to assess emerging executive function. Informed consent was obtained from the legal guardians for publishing of the images.

We paired mother-infant interactions (Figures 1A–E) and infant gut microbiome profiles (Figure 1F) collected within ±30 days of each other. Separately, we associated the early PCI data with the later behavior measured by glitter wand inhibitory control task at 19–28 months (Figure 1G). The metagenome data allowed us to resolve closely related species (e.g., *Bifidobacterium breve* vs. *Bifidobacterium longum*) and characterize their carried functional pathways (e.g., SCFA synthesis modules; tryptophan and glutamate/GABA pathways). This design enabled us to explore whether VOB under maternal unpredictability is associated with variation in gut microbial community and neuroactive metabolic gene potential.

## 2 Materials and methods

### 2.1 Cohort study participants data collection

The reported data are collected as part of a multi-site larger longitudinal cohort study designed to characterize the developmental trajectory of executive functions and its precursors from 0 to 1,000 days (Zieff et al., 2024). The relevant study population was recruited through clinics in Gugulethu, South Africa (*N* = 393). All protocols were approved by the University of Cape Town Health Sciences Faculty Research Ethics Committee (666/2021). Mothers signed informed consents on behalf of themselves and their infants. All consents and procedures were translated/communicated in the preferred family language, i.e., Xhosa in South Africa. Infants were tested in dedicated laboratory spaces over 5 time-points, beginning when infants were between 2–6 months, and roughly every 6 months after. The first testing session included parent-infant interactions, baseline EEG, a VEP assessment, collection of stool and blood samples, and structural MRI. The data reported here are shared and may be used in other publications that analyze the relationship of these variables with other collected constructs and measurements in the cohort to address novel questions. The data will not be reused to ask the same empirical questions addressed here.

### 2.2 Subsample participants and power

Of the total infants tested, 393 infants ages 2 to 6 months provided full parent-infant interaction data. Of these, videos from 263 participants were randomly-selected for hand-annotation. Four were then excluded after determining that the infants were wearing MRI ear protection during the session and were unlikely to hear the mother clearly. Three additional sessions were excluded because only one condition (see below) was recorded. One participant was not used in final analyses because age at test was not available. In all cases, the parent in the session was the mother and/or the infant’s primary caregiver. The final sample size of *N* = 255 is larger than would be needed to detect a medium sized effect (0.25) at 95% power, as determined by a G*Power analysis (*N* = 164).

### 2.3 Recording apparatus and parent-infant interaction protocol

Three tripods held 3 Logitech Webcams that connected to a computer loaded with ManyCam software. ManyCam is a software that temporarily synchronizes audio and video from multiple cameras. Mothers were seated on the floor facing infants, who were seated in car seats. One camera directly faced the infant, one faced the mother, and the final camera captured a side view of the dyad.

Mothers were instructed to play with the infant for 10 min in the same way they would at home. These 10 min were divided into two segments, one with culturally appropriate toys available (Toy condition) and another without them (No Toy condition). The order of these conditions was randomized so as to counterbalance which condition came first. Only the No Toy condition was hand annotated in the majority of participants across the two PCI testing sessions (*N* = 255 for first visit and *N* = 207 for second visit), and those data are presented here. In previous work, we found a high correlation between maternal predictability in the Toy and No Toy conditions (Forest et al., 2025a).

### 2.4 Coding of maternal sensory signals and infant visual orienting behavior

Maternal behaviors that are tactile, auditory, and visual sensory signals were coded following a coding manual developed by Davis and colleagues (Davis et al., 2017). This coding manual captures maternal sensory signals to her infant and accounts for infant eye-gaze in the context of a dyadic interaction continuously in real time. Behaviors were coded using Datavyu, a video coding and data visualization software that enables the microanalysis of behavior in dyadic interactions. Total time of video-recordings was approximately 10 min (~5 min for No Toy and ~5 min for Toy conditions). Prior to coding, video quality was assessed by evaluating camera angles, image resolution and audio clarity. In four separate passes, trained coders noted temporal onset and offset of behaviors for the following sensory categories: (1) Maternal Auditory, (2) Maternal Tactile, (3) Maternal Visual, and (4) Infant Visual. Maternal auditory signals were coded as mother vocalizing. Maternal tactile signals were coded as mother holding baby and mother touching baby. Maternal visual signals were coded as mother holds object and mother points. Each pass involved coding only a specified category of behaviors (ex. maternal verbal behaviors were coded during one pass, and in a completely separate pass, infant eye-gaze was coded) meaning all codes are entirely mutually exclusive. The onset of a behavior is defined as the first frame where the behavior occurs and offset as the last frame where the behavior occurs. Therefore, a behavior is coded for both its occurrence and duration. Identically categorized behaviors separated by less than 500 milliseconds were identified as a single event and behaviors separated by 500 milliseconds or more or categorized differently were annotated as separate events. Instances where mother and/or infant behaviors were not codable because they were not clear in the video or audio (i.e., instances where mother went out of view to grab a new toy; instances where experimenter vocal input hinders clarity on what mother is saying) were also annotated and classified as unknown. This coding approach allows for the dyadic interactions and relationship of behaviors to be explored solely on the basis of co-occurrence in real time and not coder interpretation of contingency.

Each coded video underwent the following process: (1) primary coding (2) reliability coding, where a different, secondary coder codes the same ~10-min interval in a subset of videos and (3) reliability check, where a script computes the percent reliability of each column. If interobserver reliability was below 80%, the indicated coders revised their coding separately for that participant and the reliability check process was repeated until interrater reliability criteria of at least 0.8 were met for every maternal behavior.

### 2.5 Measuring maternal unpredictability/entropy & infant visual attention orienting behavior

The coding process resulted in data points displayed as cells, each representing an interval in time. If a behavior was coded as “active” during the given interval, the corresponding cell was assigned a value of 1; otherwise, cells were filled in with 0 s. For each participant, data include temporally aligned columns representing the onset and offset of each occurrence of any of the 5 maternal behaviors (vocalizing, holding baby, touching baby, holding object, pointing), as well as for infant gaze toward the mother or the object mother is in contact with. Because multiple maternal behaviors can happen simultaneously, each time a new instance of any of the five targeted behaviors is coded as active, a new behavioral state is generated, with the new state’s onset being the previous state’s offset. We operationalize discrete independent behavioral states as any combination of the 5 maternal behaviors. For example, a mother might vocalize and hold an object at the same time.

Broadly, Shannon Entropy calculations involve the Markov property, where the predictability of any one maternal behavioral state (*n* + 1) is conditioned only on the immediately previous state (*n*). If we define X, Y as two discrete random variables, X being the initial state and Y being the final state of any two-state transitions in a stochastic process, we have the conditional entropy formula of Y given X as:


$$\begin{array}{c} H(Y|X)=-\sum_{x\in X,y\in Y}p(x,y)\log(p(x,y))+\sum_{x\in X,y\in Y}p(x,y)\log(p(x))\\ =H(X,Y)-H(X) \end{array}$$

Here p(x) denotes the proportion of times that a particular value of X happens out of all X occurred values in the data set. Similarly, p(x,y) denotes the joint probability of the particular values x, y happening together. For processing the data, we used the Python programming language (version 3.9) (Van Rossum and Drake, 2009) via Jupyter Notebook (Thomas et al., 2016), a web-based interactive computing platform.

Visual attention orienting behavior was coded in a separate pass from maternal behaviors. We calculated here the number of shifts in looking to/from the mother, defined as the number of times annotators indicated that infants clearly returned their gaze to the mother after having looked away. We corrected this value for total time on task for each dyad.

### 2.6 Glitter wand inhibitory control task

The child is seated in a caregiver’s lab in front of a small table. The Experimenter shows the child the glitter wand, flash the lights, and move it around. The Expermenter attracts the child’s attention by saying, “look at this wand! Look at its lights!”. They then place the glitter wand on the table within the child’s reach, looks at the child and says “Don’t touch this toy until we say it is okay. You can play with the toy if you can wait.” The Experimenter looks away from the child and starts a timer once hands have been released from the toy. If 30 s passes and the child has not yet touched it, the Experimenter says, “Great job! You didn’t touch it, so now you can play with the toy for a bit!” If the child touches the glitter wand, the timer is stopped and the Experimenter says, “It’s okay; you can touch it now.” The score is the wait time from the release of the glitter wand to the time the child touches it.

### 2.7 Biospecimen sample collection and DNA extraction

Stool samples were collected at the clinic by a research assistant, who directly transferred them from the diaper to the Zymo DNA/RNA Shield™ Fecal Collection Tube (#R1101, Zymo Research Corp., Irvine, USA) and immediately froze them at −80 °C. Samples were not collected if the subjects had taken antibiotics within the preceding 2 weeks. The DNA extraction process was conducted at the Medical Microbiology Department of the University of Cape Town, South Africa. The samples were processed using the Zymo Research Fecal DNA MiniPrep kit (#D4300, Zymo Research Corp., Irvine, USA) according to the manufacturer’s instructions. ZymoBIOMICS® Microbial Community Standards (#D6300 and #D6310, Zymo Research Corp., Irvine, USA) were used as controls and extracted in the same manner as the stool samples. The DNA yield and purity were measured with a NanoDrop® ND-2000 spectrophotometer (Thermo Scientific, Nanodrop Technologies Inc., Wilmington, USA). Library preparation protocols were the same across all samples included in the study.

### 2.8 Sequencing

All samples underwent shotgun metagenomic sequencing at the Integrated Microbiome Research Resource (IMR) at Dalhousie University, Nova Scotia, Canada. A pooled library, accommodating up to 96 samples per run, was prepared using the Illumina Nextera Flex Kit for MiSeq and NextSeq from 1 ng of each sample. The samples were then pooled onto a plate and sequenced on the Illumina NextSeq 2000 platform with 150 + 150 bp paired-end P3 cells, generating 24 million raw reads and 3.6 Gb of sequence per sample (Comeau and Filloramo, 2023). All samples were sequenced in a single batch.

### 2.9 Metagenome processing

Raw metagenomic sequence reads were processed using tools from the bioBakery as previously described (Beghini et al., 2021; Bonham et al., 2023). Briefly, KneadData v0.10.0 was used with default parameters to trim low-quality reads and remove human sequences (using reference database hg37). Next, MetaPhlAn v3.1.0 (using database mpa_v31_CHOCOPhlAn_201901) was used with default parameters to map microbial marker genes to generate taxonomic profiles. Taxonomic profiles and raw reads were passed to HUMAnN v3.7 to generate stratified functional profiles.

### 2.10 PCI-microbiome dataset composition

A paired dataset for analysis of microbial features and Parent–Child Interaction (PCI) outcomes was built from participants who had both metagenomes and recorded/processed videos. Participants were preliminarily eligible for inclusion if the stool sample was collected less than 30 days apart from the video recording. For every preliminarily eligible participant, final inclusion occurred for every combination of (1) a QC-passed sequencing run that yielded a profiled metagenome; and (2) a complete-set of metadata containing the age in days at the time of stool collection, child sex (assigned at birth), maternal education (in years of formal education, and included as a proxy of socioeconomic status), 2-category delivery mode (vaginal, C-section), and 3-category infant feeding practice (exclusive breastfeeding, mixed feeding, or formula feeding), alongside the paired maternal unpredictability and VOB. Microbial community profiles - both taxonomic and functional - were represented as matrices of feature abundances by samples (rows). The final sample size for the paired microbial dataset was *n* = 88 samples.

### 2.11 Stool sample exclusion criterion

One sample was excluded *post hoc* after analysis initially found a significant association between *Streptococcus mitis* abundance and infant VOB. Further inspection revealed that this association was driven by a single outlier with both a high VOB and an abnormally high *S. mitis* relative abundance (>50%). *S. mitis* is typically an oral and respiratory commensal rather than a gut resident, where its presence is linked to dysbiosis and potential pathogenicity (Abdelbary et al., 2022). This sample exerted excessive leverage on the linear model (Cook’s D>>3×mean(D) ), artificially inflating the effect. Excluding it rendered the *S. mitis*-VOB association non-significant while leaving all other results unchanged. A short sensitivity supplement to support sample exclusion is included in Supplementary Figure S1 and Supplementary Table S1, and, for transparency, data for this sample is included in the Data Availability and Reproducibility statement. As a consequence, the resulting dataset for analysis consisted of *n* = 87 paired metagenomes and PCI outcomes.

### 2.12 Exploratory microbial community analysis

Principal coordinates analysis was performed in the Julia programming language v1.12.1 (Bezanson et al., 2012) using the Microbiome.jl package v0.10.0 (Bonham et al., 2021). Bray–Curtis dissimilarity was calculated across all pairs of samples, filtering for species-level classification, with Distances.jl v0.10.12. Classical multidimensional scaling was performed on the dissimilarity matrix using MultivariateStats.jl v0.10.3 and PERMANOVA.jl v0.1.1 (Newbury, n.d.). Additional PERMANOVA analyses and variance partitioning were performed with the vegan package v2.6.8 (Oksanen et al., 2026) in the R language v4.5.2 (R Core Team, 2021).

### 2.13 Control factors and significance framework for microbial analyses

All microbiome-associated statistical analyses were conducted under a consistent modeling framework with a prespecified set of covariates. Across all microbial models, including linear models, machine learning approaches, and feature set enrichment analyses (FSEA), we included the following covariates to account for key biological sources of variation: (1) infant age at stool collection, (2) infant sex, (3) maternal education, (4) delivery mode, and (5) feeding practice. These variables were selected *a priori* based on their established roles as determinants of infant gut microbiome composition and developmental trajectories. Multiple hypothesis testing in microbial association analyses was controlled using the Benjamini-Hochberg false discovery rate (FDR) procedure, with a prespecified significance threshold of *q* < 0.2 applied uniformly across all analyses. All reported associations are presented with corresponding effect sizes and exact *p*-values, enabling full assessment of result strength.

### 2.14 Linear modeling protocol

Individual taxonomic features were assessed for associations with VOB features using multivariable linear models according to the MaAsLin v3 methodology (Nickols et al., 2026) implemented using the GLM.jl package v1.8.3 (Bates et al., 2023). The equation tested was species ∼ VOB + control factors *(See Methods 2.13)* where species is the arcsin-normalized relative abundance of each taxon when present in the sample and VOB is the unit-range-normalized numerical value of the VOB feature. FDR correction was employed via a combination of HypothesisTesting.jl v0.11.6 and MultipleTesting.jl v0.6.0 (Gehring, 2023).

### 2.15 Nonlinear modeling protocol

Random Forests (RFs) (Breiman, 2001) were selected as the nonlinear prediction engine and processed using the DecisionTree.jl v0.12.4 (Sadeghi et al., 2022) implementation, inside the MLJ.jl v0.19.2 (Blaom et al., 2020) framework. Models were trained and benchmarked with a repeated cross-validation approach with different random number generator (RNG) seeds. One hundred repetitions of threefold cross-validation (CV) with 10 different intra-fold RNG states each were used, for a total of 3,000 experiments per input set. RF hyperparameters were optimized from a grid; gridpoint details are available alongside the data reproducibility notebooks. After the training procedures, the RMSE for VOB, along with Pearson’s correlation coefficient (R), were benchmarked on the validation sets for the winning combination of hyperparameters.

### 2.16 Feature set enrichment analysis protocol

To assess whether microbial neuroactive pathways varied across combinations of maternal unpredictability and infant VOB, we performed feature set enrichment analysis (FSEA) on metagenomic functional profiles. Specifically, we tested whether the association between microbial gene content and VOB depended on the environmental context of maternal unpredictability.

For each UniRef90 gene family, binary gene presence/absence was modeled using logistic regression implemented in GLM.jl. Models included pre-specified covariables (see Methods 2.13), together with infant VOB, maternal unpredictability (MatEnt), and their interaction term:


$$\begin{array}{l} p_{i}=\\ \frac{1}{1+e^{-(\beta_{0}+\beta_{1}\mathrm{Age}+\beta_{2}\mathrm{Sex}\;\;+\beta_{3}\text{MatEdu}+\beta_{4}\text{Feeding}+\beta_{5}\text{Delivery}+\beta_{6}\text{MatEnt}+\beta_{7}\mathrm{VOB}+\beta_{8}\text{MatEnt}\times \mathrm{VOB})}} \end{array}$$

where $p_{i}$ ​ represents the probability of observing a given UniRef90 gene family in sample i .

This interaction framework was used to evaluate whether microbial associations with VOB differed across levels of maternal unpredictability after accounting for major biological determinants of infant gut microbial composition. Following regression, genes were grouped into previously curated neuroactive metabolic gene sets (Valles-Colomer et al., 2019). For each model term of interest, genes were ranked according to the ratio between the parameter estimate and its standard error. Enrichment scores (ES) were then calculated for each neuroactive gene set using Wilcoxon rank-sum tests to assess enrichment or depletion within the ranked distributions.

## 3 Results

### 3.1 Unpredictable caregiving predicts a mature-for-age visual orienting behavior strategy

We analyzed 255 mother-infant dyads (119 female infants, 136 male infants), who participated in a semi-naturalistic interaction without toys or structured prompts at a testing site in Cape Town, South Africa (Figures 1A,B). Three cameras captured the mother, the infant and the dyad. Videos were hand-annotated (Figure 1) for this analysis according to a validated coding scheme (Davis et al., 2017; Vegetabile et al., 2019). Sample sizes and descriptive statistics for all measures are listed in Table 1.

**Table 1:** Sample demographics for *N* = 255 infants who contributed full dyadic interaction and Session 1 age data.

Metadatum	*N*	Minimum	Maximum	Mean	SD
Dyadic Interaction Session 1 Infant Age (days)	255	60	176	113.53	25.85
Dyadic Interaction Session 2 Infant Age (days)	207	138	364	261.71	44.36
Inhibitory Control Task Age (days)	194	578	828	653.21	41.66
Microbiome Collection Age (days)	87	65	163	110.64	23.01
Maternal Education (years)	255	7	15.5	11.74	1.39
Maternal Depression (EPDS score)	235	0	22	6.63	5.82

We first examined whether maternal unpredictability during the interaction was associated with shifts in infant visual orienting behavior (VOB), defined as the number of gaze shifts to or from the mother, the primary source of information during the dyadic interaction. Based on prior work suggesting that infants increase sampling of alternative information sources under unpredictable conditions (Kidd et al., 2012), we hypothesized that higher maternal unpredictability would be associated with more frequent gaze shifts. However, VOB shifts, to the extent that they reflect oculomotor control, can be slow to develop, with very early infancy marked by rapid change and growth in oculomotor control (Johnson et al., 2015). Therefore, showing higher rates of VOB shifts, in response to unpredictable maternal behavior, may reflect a mature-for-age pattern in infants in our 2–6 month-old infant sample.

Maternal unpredictability was positively correlated with infant VOB (*r* = 0.35, *p* = 1 × 10^−8^; Table 2). Maternal unpredictability was not significantly associated with maternal education or depression. To determine that the association between maternal unpredictability and VOB was not driven by infant age, we conducted an ANCOVA with VOB as the dependent variable, with infant age (in days) as a covariate and maternal unpredictability as a continuous variable. VOB shifts increased with infant age, as expected based on the developmental visual orienting literature (Amso and Scerif, 2015), *F*(1,252) = 6.483, *p* = 0.011, ηp^2^ = 0.025. VOB shifts also independently increased with higher values of maternal unpredictability, while controlling for infant age in the model, *F*(1,253) = 29.099, *p* = 2 × 10^−7^, ηp^2^ = 0.103.

**Table 2:** Correlations of maternal unpredictability.

Row × column	Maternal unpredictability	Infant VOB	Infant Age Session 1	Maternal education
Infant VOB	0.35** (*N* = 255)			
Infant Age Session 1	0.21** (*N* = 255)	0.22** (*N* = 255)		
Maternal Education	0.006 (*N* = 255)	−0.06 (*N* = 255)	0.05 (*N* = 255)	
Maternal Depression (EPDS)	−0.01 (*N* = 235)	0.02 (*N* = 235)	−0.04 (*N* = 235)	0.001 (*N* = 235)

**p = 0.001.

**Figure 2:**
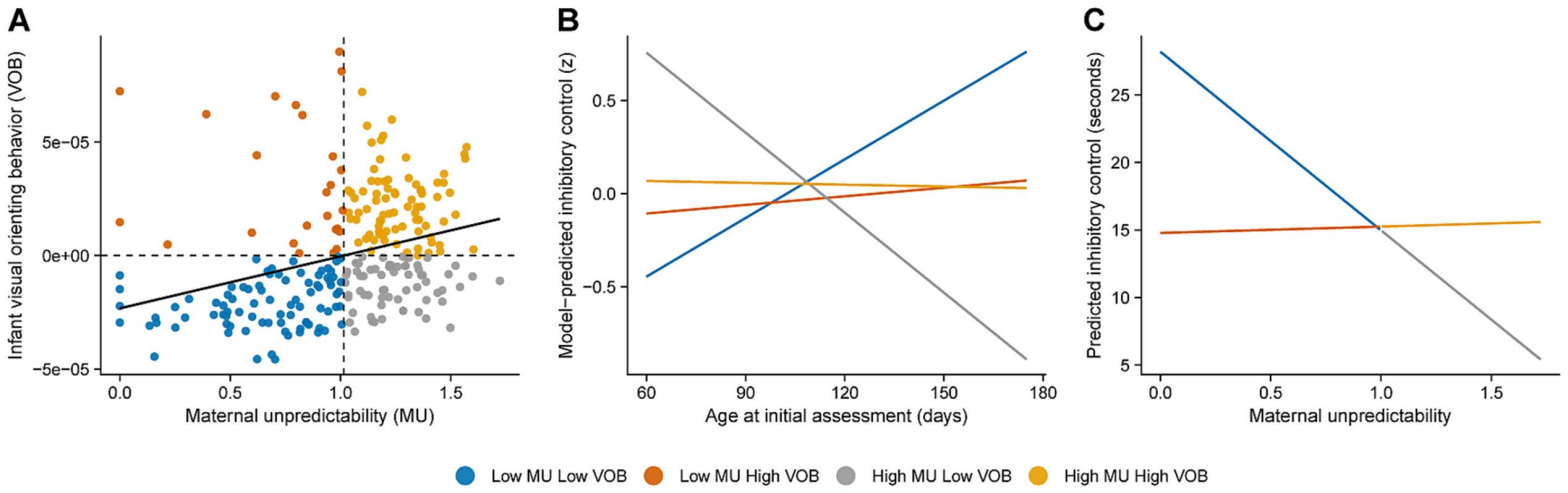
Maternal unpredictability, infant VOB, and later inhibitory control. **(A)** Association between maternal unpredictability (MU) and infant visual orienting behavior (VOB) shifts (residual values for age) in 2–6 month-old infants. Higher VOB indicates higher values than expected for age. **(B)** Simple slopes illustrate the relationship between the four groups delineated in panel A and inhibitory control task performance when the same infants were 19–28 months-old. **(C)** Simple slopes for maternal unpredictability predicting inhibitory control separately when infants are tested in the upper end of the age range.

To test whether infants exposed to more unpredictable caregiving had a higher VOB *for their age*, we extracted the residuals from a model that examined only the effects of infant age on VOB, *F*(1,253) = 13.017, *p* = 4 × 10^−4^, ηp^2^ = 0.049. Age-adjusted VOB remained positively associated with maternal unpredictability [*r*(253) = 0.314, *p* = 3 × 10^−7^; Figure 2A]. Thus, infants whose mothers were more unpredictable had more frequent gaze shifts than would be expected based on age alone.

An examination of the data pattern in Figure 2A suggested that VOB shifts were not uniformly distributed across maternal unpredictability continuum. Infants exposed to low maternal unpredictability showed relatively slower-for-age VOB developmental patterns (*n* = 25 high VOB and *n* = 85 low VOB), or fewer VOB shifts, while infants exposed to higher unpredictability showed more variability in VOB responses for age (*n* = 80 high VOB and *n* = 65 low VOB). We color-coded each mother-infant dyad on the two variables of interest for illustration: infant VOB *for age* (mean split to create high/low VOB groupings) and unpredictability (mean split to create high/low maternal unpredictability groupings). VOB shifts are not uniformly distributed across maternal unpredictability groupings (Figure 2A). A Pearson chi-square test confirmed that the distribution significantly differed across groups [(1, *N* = 254) = 27.18, *p* = 2 × 10^−7^]. These findings suggest that maternal unpredictability is associated with increased variability in visual attentional strategies.

### 3.2 Persistence across time

To verify persistence of effects, we tested the subset of dyads using the same unstructured dyadic interaction protocol 3 months after their first session (see Table 1). Maternal unpredictability showed correlation across time points [*r*(206) = 0.162, *p* = 0.02], consistent with this behavior reflecting a trait-like maternal characteristic. At the second timepoint, maternal unpredictability was again positively correlated with VOB [*r*(206) = 0.194, *p* = 0.005], replicating the concurrent associations observed during the first timepoint. Furthermore, maternal unpredictability at the early testing time was not correlated with VOB at the later time, and later maternal unpredictability was not correlated with VOB at the earlier testing time (all *ns*), suggesting that maternal unpredictability and VOB were coupled within timepoints, but did not predict one another longitudinally. Taken together, these results demonstrate that increased VOB in response to maternal unpredictability reflects a context-sensitive visual orienting, moment-to-moment strategy to manage learnability of input, rather than a driver of general disorganized visual attention orienting development in infancy.

### 3.3 High infant VOB in the context of high maternal unpredictability is associated with resilience in later inhibitory control scores

Davis et al. (2019) showed that high maternal unpredictability in infancy was associated with poor effortful control in childhood. We began by asking whether, in this South African cohort, we could replicate the negative relationship obtained with control and maternal unpredictability in Western cohorts. A subset of infants completed an inhibitory control task when they were between 19–28 months of age (*n* = 194, 90 female). Participants were presented with a shiny, glittery toy and instructed to wait up to 30 s before approaching the toy for play. The inhibitory control was indexed as wait time (in seconds). This score was not correlated with inhibitory control task test age [*r*(194) = 0.069, *p* = 0.34].

A general linear model predicting inhibitory control included maternal unpredictability, infant VOB (residual for age), and infant age at initial assessment (and their interactions), with infant sex as a covariate. There was no significant main effect of sex, *F*(1, 185) = 0.548, *p* = 0.460, ηp^2^ = 0.003. We observed trend-level main effects of infant age, *F*(1, 185) = 3.60, *p* = 0.059, ηp^2^ = 0.019, and VOB, *F*(1, 185) = 3.312, *p* = 0.069, ηp^2^ = 0.018, and a significant main effect of maternal unpredictability, *F*(1,185) = 4.118, *p* = 0.043, ηp^2^ = 0.022. Interactions were as follows: age by maternal unpredictability, *F*(1, 185) = 4.478, *p* = 0.035, ηp^2^ = 0.024; age by VOB, *F*(1, 185) = 3.151, *p* = 0.078, ηp^2^ = 0.017; maternal unpredictability by VOB, *F*(1,185) = 4.079, *p* = 0.044, ηp^2^ = 0.022; and an age by maternal unpredictability by VOB three-way interaction, *F*(1, 185) = 4.056, *p* = 0.045, ηp^2^ = 0.022. Higher maternal unpredictability was associated with lower inhibitory control scores. The interaction with age indicates that assessments conducted in infants that were older in the 2 to 6 month age range were more likely to reveal this effect.

To interpret the patterns revealed by the maternal unpredictability by age by VOB three-way interaction, we examined the simple slope of maternal unpredictability at ±1 SD of infant age and VOB (covariates fixed). At the higher end of the 2–6 month age range (+1 SD ≈ 139.0 days ≈ 4.56 months), higher maternal unpredictability in the context of low infant VOB (grey markers in Figure 2A) predicted shorter wait times (poorer inhibitory control), *b* = −1.027, *SE* = 0.399, *p* = 0.016 (HC3 *p* = 0.010). In contrast, when VOB was high for age (+1 SD; yellow markers in Figure 2A), the maternal unpredictability slope was indistinguishable from zero: *b* = +0.039, *SE* = 0.622, *p* = 0.940 (HC3 *p* = 0.949). The difference between the two slopes (Low − High VOB) was sizable (*Δb* = −1.066, *SE* = 0.747). At the younger end (−1 SD ≈ 87.9 days ≈ 2.89 months), maternal unpredictability slopes were not significant whether VOB was low (*b* = −0.678, *SE* = 0.458, *p* = 0.105) or high (*b* = +0.167, *SE* = 0.396, *p* = 0.629). This pattern suggests that higher infant VOB, than would be expected for age, attenuates the negative association between maternal unpredictability and later inhibitory control, particularly at older infant ages in our range. We next explored whether individual differences in VOB shifts in early infancy are associated with gut microbiome composition and function.

### 3.4 Microbiome composition is associated with the VOB strategy

We examined microbiome data for evidence of association between microbial composition and VOB. Data on both the gut microbiome and maternal–infant interactions were available for *n* = 87 (37 female) of the same infants whose data are discussed above. Stool samples (*n* = 87, one per infant) were collected for metagenomic analysis concurrently (± 30 days) with PCI measurements. We used this dataset to analyze the effect of various factors on gut microbial community diversity at the taxonomic and functional gene levels. Specifically, we performed both taxonomic and functional profiling of the metagenomes using a uniform computational pipeline (*see Materials and Methods 2.7–2.10*). Taxonomic profiles consisted of species-level relative abundances (with an average 34 [*SD* = 14] species per sample). Functional profiles consisted of relative abundances of gene families and pathways (with an average 274,200 [*SD* = 113,100] gene functions per sample).

**Figure 3:**
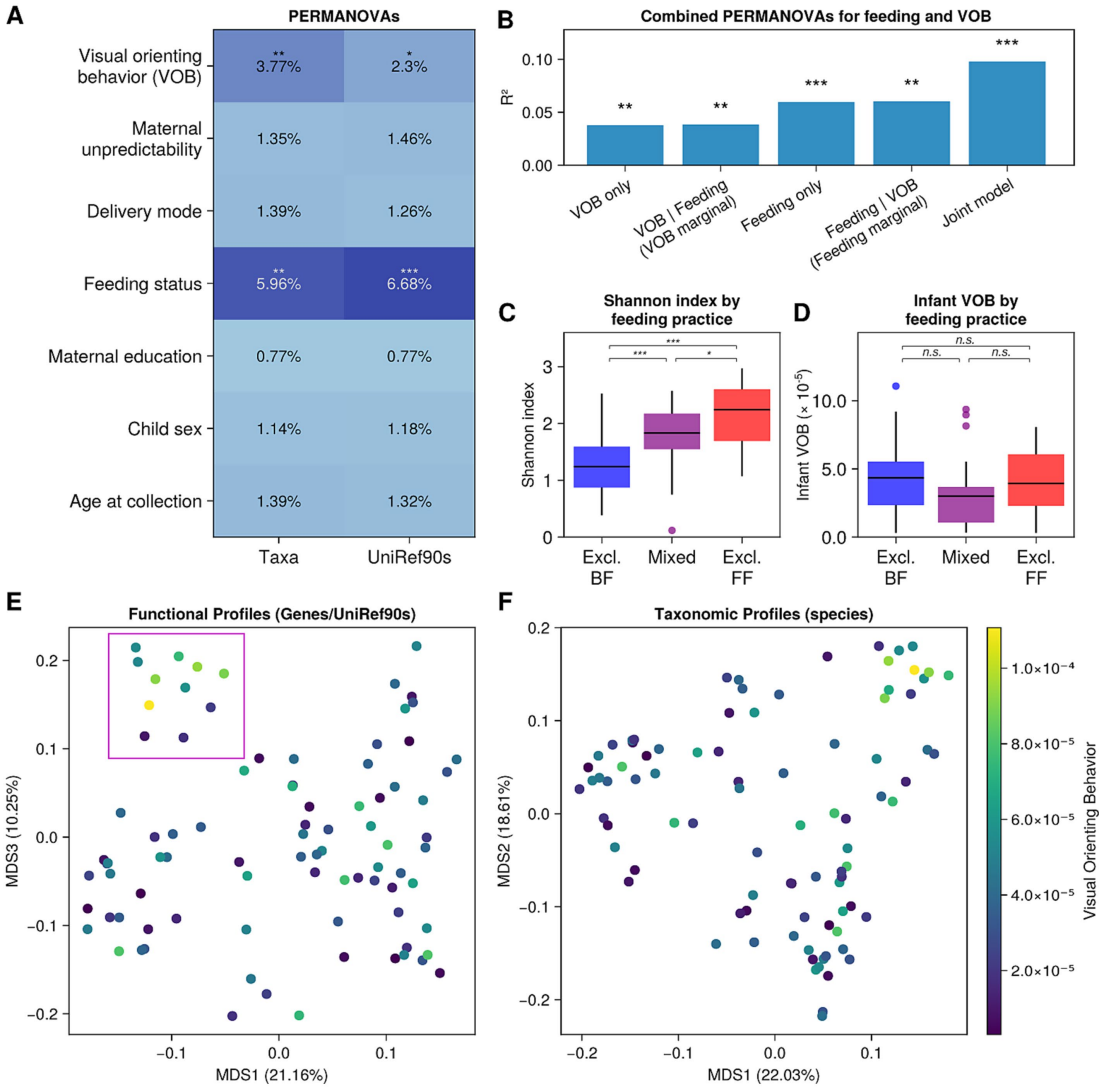
Infant VOB and microbial community profiles share variance explained at both taxonomic and functional levels. **(A)** Permutational multivariate analysis of variance (PERMANOVA) testing associations between infant metadata variables and gut microbial taxonomic (species-level relative abundances) and functional profiles (UniRef90 gene function relative abundances). Values indicate the percentage of variance explained (R^2^). Significance levels are indicated as follows: *p < 0.05; **p < 0.01; ***p < 0.001. **(B)** Combined PERMANOVA models evaluating the contribution of VOB and feeding status to microbiome variation. **(C)** Shannon diversity index across feeding groups, including exclusively breastfed (excl. BF), mixed-fed, and exclusively formula-fed (Excl. FF) infants. Groups differing significantly in alpha diversity are indicated as follows: *p < 0.05; **p < 0.01; ***p < 0.001. **(D)** Infant VOB values across feeding groups. No significant differences (N. S.) are observed between feeding categories. **(E)** Principal coordinate analysis (PCoA) by multidimensional scaling (MDS) of Bray-Curtis dissimilarities of functional profiles (Uniref90 gene functions). The percent variance explained by each principal coordinate is indicated on the X and Y axes. Samples (dots) are colored *a posteriori* by infant visual attention shifts. The high-attention cluster mentioned in the results is highlighted. **(F)** Principal coordinate analysis (PCoA) by multidimensional scaling (MDS) of Bray-Curtis dissimilarities of taxonomic profiles (species).

We began with a permutational analysis of variance (PERMANOVA), intended to preliminarily assess whether specific metadata could explain variance in the inter-sample diversity (*β*-diversity) of gut metagenomes. β-diversity was computed as pairwise Bray-Curtis dissimilarities between metagenomes. While factors such as age at collection, infant sex, maternal education, maternal unpredictability, and delivery mode did not significantly explain variance in community diversity (*p* > 0.05), two factors stood out as significant: infant feeding practices (R^2^ = 0.060, *p* = 0.001 for taxa; *R*^2^ = 0.067, *p* = 0.001 for genes; Figure 3A) and VOB (*R*^2^ = 0.038, *p* = 0.002 for taxa; *R*^2^ = 0.023, *p* = 0.012 for genes; Figure 3A).

### 3.5 Explanatory structure of VOB is orthogonal to feeding practices

Because feeding practices are among the strongest known effectors of early-life gut microbial assembly, we sought to determine whether the observed group-effect associations with VOB could instead be explained by (and tracking on) underlying feeding-related effects. To address this, we performed additional PERMANOVA and variance partitioning analyses comparing the independent and joint contributions of feeding status and VOB to *β*-diversity variation.

In a multivariable PERMANOVA analysis combining both factors and looking for marginal effects, VOB remained independently associated with taxonomic β-diversity even after accounting for feeding status (*marginal-to-feeding* R^2^ = 0.038, *p* = 0.001; Figure 3B), with a largely unchanged effect size relative to the unadjusted model. This was also true for infant feeding, whose effect marginal to VOB was also unchanged relative to the unadjusted model (*marginal-to-VOB* R^2^ = 0.060, *p* = 0.001; Figure 3B). Similarly, at the functional level, VOB retained a significant independent contribution after conditioning on feeding (*marginal-to-feeding* R^2^ = 0.022, *p* = 0.010; Figure 3B). As expected, joint models including both feeding status and VOB explained more variance than either variable alone at both the taxonomic (R^2^ = 0.098, *p* = 0.001) and functional levels (R^2^ = 0.089, *p* = 0.001; Figure 3B).

To further quantify the degree of overlap between feeding-associated and VOB-associated microbial variation, we performed variance partitioning analyses using distance-based redundancy analysis (dbRDA). At the taxonomic level, feeding status and VOB explained largely distinct fractions of *β*-diversity variation, with minimal shared explanatory variance between predictors (shared adjusted R^2^ ≈ 0). Specifically, the unique contribution of VOB after accounting for feeding status was comparable to that of feeding after accounting for VOB (adjusted R^2^ = 0.028 and 0.039, respectively). Permutation testing of the partial dbRDA models confirmed that both the VOB-associated fraction (*F* = 3.52, *p* = 0.001) and the feeding-associated fraction (*F* = 2.77, *p* = 0.002) were independently significant.

A similar pattern was observed at the functional level. Variance partitioning revealed essentially no shared variance between VOB and feeding status in UniRef90 functional profiles (shared adjusted R^2^ ≈ 0), with both predictors retaining their previous explanatory potential after conditioning on the other. The unique fraction associated with VOB remained significant in partial dbRDA analyses (*F* = 2.02, *p* = 0.006), as did the unique fraction associated with feeding status (*F* = 3.01, *p* = 0.001). Consistent with the variance partitioning analyses, feeding groups exhibited significant differences in microbial *α*-diversity (Shannon index, Figure 3C), while infant VOB did not significantly differ across feeding categories (Figure 3D), arguing against feeding status as a primary driver of the observed VOB-associated microbial signatures.

Together, these analyses indicate that the microbial signatures associated with VOB are not secondary to canonical feeding-associated microbial variation. Instead, feeding status and VOB explain orthogonal components of microbiome structure and function, supporting the interpretation that infant attentional strategies are associated with a distinct axis of gut microbial variation beyond the established dietary determinants of early-life microbial assembly.

### 3.6 High VOB participants are unevenly distributed in *β*-diversity topology

Group-effect analyses provided an initial indication that infants with high VOB may have a distinct gut-microbial signature independent of other key early-life factors such as age. To further explore this hypothesis, we conducted ordination of the β-diversity matrix of functional gene profiles using multidimensional scaling (MDS). This approach allows us to reduce complex, high-dimensional diversity patterns into a few interpretable axes that capture the major sources of variation across samples. The first three principal coordinates explained 45.86% of the variance. While the primary projection of PC1 vs. PC2 showed no appreciable patterns (Supplementary Figure S2), the projection of PC1 vs. PC3 (Figure 3E) revealed that children with higher VOB shifts form a visually distinct cluster.

To formally assess the clustering patterns beyond the visual readout, we applied k-means clustering. We first determined the optimal number of clusters (*k*) using unsupervised statistical methods. A combination of Within-Cluster Sum of Squares (WCSS) and Silhouette Scores (Supplementary Figure S3A), irrespective of VOB, resulted in optimal *k* = 4. We then proceeded to compare the values of VOB between the resulting clusters. Consistent with the visual separation in Figure 3B, one cluster stood out with a mean infant VOB nearly twice as high as the others. While most clusters had mean VOB values ranging from 3.31 × 10^−5^ (*SD* = 1.98 × 10^−5^) to 3.78 × 10^−5^ (*SD* = 2.08 × 10^−5^), this group exhibited a mean of 5.79 × 10^−5^ (*SD* = 3.48 × 10^−5^), significantly different from the others (*p* = 0.002; Supplementary Figures S3B,C).

To determine whether taxonomic β-diversity patterns would mimic the VOB distribution patterns found in the functional gene data, we replicated the MDS ordination analysis using species-level data. The first three principal components explained 50.11% of the variance in taxonomic-level β-diversity (Figure 3F). While the projected sample scores did not exhibit the same distinct structure, the principal coordinates revealed that a small subset of microorganisms drove variation in community diversity. PC1 (22.02% of total variance) was primarily associated with typically exclusive infant enterotypes dominated by either *Bifidobacterium longum* (*R*^2^ = 0.837) or *Bifidobacterium breve* (R^2^ = 0.318). Notably, PC1 was weakly correlated with VOB (*R*^2^ = 0.103), despite VOB not being included as a factor in the ordination analysis. PC2 (18.61% of total variance) was associated with a balance between prominent *Bifidobacterium* spp. (*B. longum*, R^2^ = 0.078; *B. breve*, R^2^ = 0.522; *Bifidobacterium bifidum*, *R*^2^ = 0.168) and other genera commonly found in the developing guts of infants (*Ruminococcus gnavus*, *R*^2^ = 0.330; *Erysipelatoclostridium ramosum*, *R*^2^ = 0.165; *Escherichia coli*, *R*^2^ = 0.176; Supplementary Figure S4).

### 3.7 VOB variation is associated with *Bifidobacterium* spp

**Figure 4:**
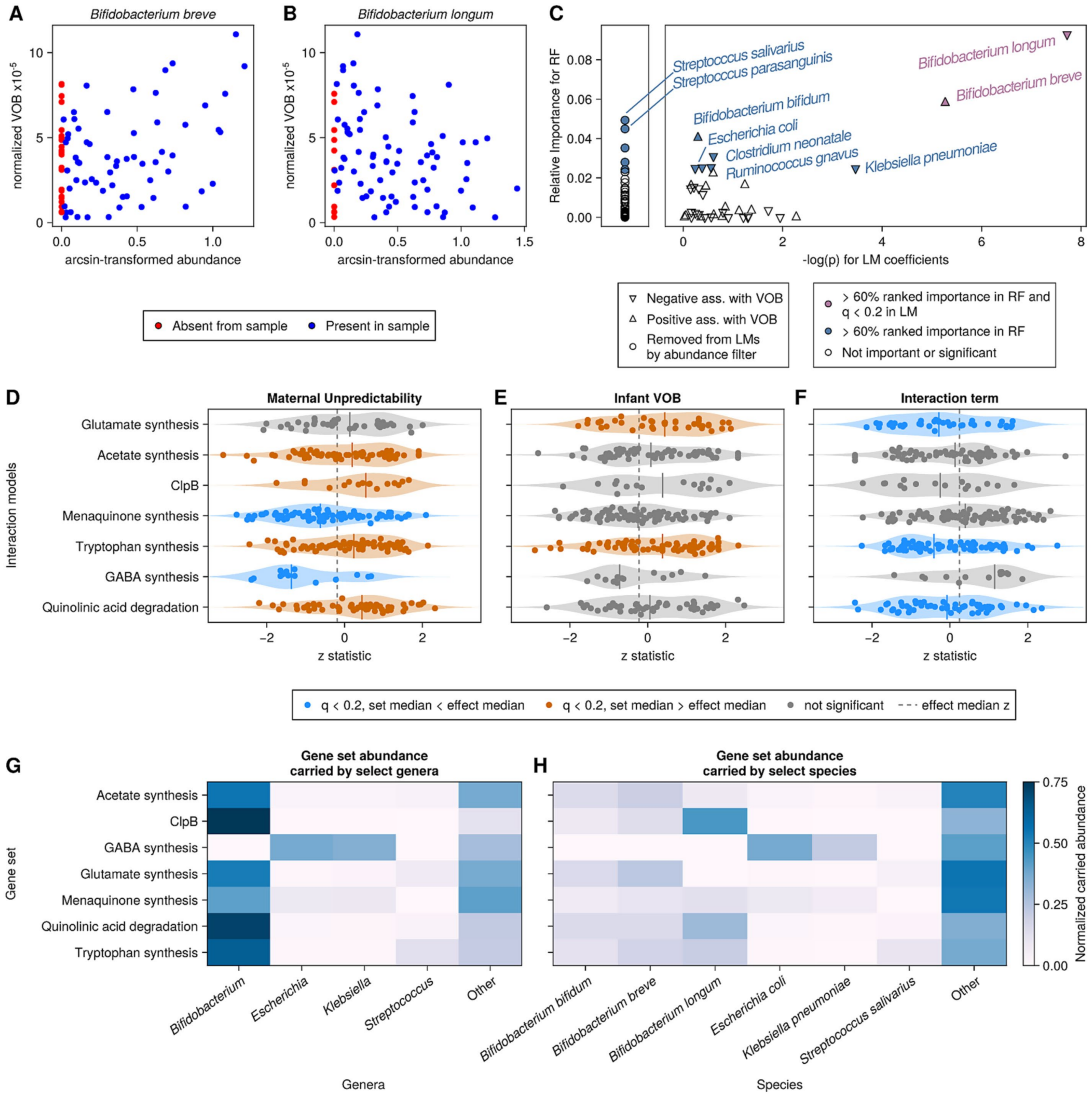
Microbial gene functions and their carrier species are associated with infant VOB. Unit-range normalized VOB as a function of arcsin-normalized species abundance for both FDR-passing microbes, *B. breve* **(A)** and *B. longum* **(B)**. Colors indicate whether the microbe was detected in the profiled metagenome. **(C)** Comparison between the random forest feature importance (measured as mean decrease in impurity - MDI) and unique effect significance (*p*-value) for each input species toward the prediction of infant VOB. Main panel marker shapes indicate whether species were positively or negatively associated with the outcome on linear models. The side panel shows species that were removed from linear models by the abundance filter, which only have an RF importance associated. **(D,E,F)**Feature set enrichment analysis (FSEA) of microbial neuroactive genes and maternal unpredictability (D), infant VOB (E), and their interaction (F). FSEA results are shown for neuroactive gene sets where at least one tested component, maternal unpredictability, infant visual attention, or their interaction, had a significant hit (*q* < 0.2). Dots indicate the *Z*-statistic from logistic regression for each gene in a gene set; vertical bars indicate the median *Z*-statistics for the whole gene set. **(G,H)**Proportion map of carriers for each characterized gene function on each neuroactive gene set shown on Panels (D,E,F). (G) Gene set abundance carried by select genera; (H) Gene set abundance carried by select species. Shade strength indicates the proportional count of detected genes, in each gene set, carried by taxa in columns.

Building on the ordination patterns and exploratory data analysis, we tested individual species for associations with maternal unpredictability and VOB. We modeled normalized VOB as an outcome for each microbial feature (*m* = 36) that met a minimum prevalence of 5% and a mean relative abundance ≥ 1%, while controlling for the relevant variables (*see Methods 2.13–2.14*). Microbial relative abundances were arcsine-transformed. No microbial species showed a significant association with maternal unpredictability after false-discovery rate (FDR) correction using the Benjamini-Hochberg method. However, for VOB, *B. longum* (*β* = −0.611, *p* = 0.0004, *q* = 0.016) and *B. breve* (*β* = 0.556, *p* = 0.005, *q* = 0.09) remained significant after FDR correction (Figures 4A,B).

While individual associations provide insights into effect sizes and significance, gut microbial community effects should be considered within their broader compositional context. To address this, we used a nonparametric multivariable model, Random Forest (RF), to predict VOB from all microbial features that met the 5% prevalence filter, regardless of mean abundance (*m* = 138), again controlling for the relevant variables*. In a three-fold-cross-validation setting, the model achieved a Root Mean Square Error (RMSE) of 0.263 and a Pearson correlation of 0.240 between the predicted and ground-truth unit-range normalized VOB in validation sets. We then extracted the variable importance ranking from the RF model and compared it to the significance of effects from linear models. As expected, both *B. breve* and *B. longum* ranked highest in both scales, even surpassing the infant age at sample collection feature (Figure 4C). Additionally, *Klebsiella pneumoniae* achieved high ranks for both importance and effect size significance. A third group, including species such as *B. bifidum*, and *E. coli*, showed high importance in RF models despite showing modest effects in linear models. Species that did not pass prevalence and abundance filters for inclusion in linear models also ranked high in RF importance, specifically, *Streptococcus salivarius, Streptococcus parasanguinis, and Enterococcus faecalis*.

Additionally, we probed whether microbial features would improve the prediction of VOB alongside maternal unpredictability. To do so, we built a second RF model predicting VOB from a combination of Maternal Unpredictability and 30 top-ranking microbial features. Predictions achieved a Pearson R of 0.386 with the ground truth, which, despite being a marginal numerical improvement over the prior correlation between VOB and Maternal unpredictability (0.380), was not statistically significant with the sample size (*p* > 0.05, *n* = 87). Overall, predictive performance was modest, indicating that the predictive potential of microbial taxa toward VOB is subtle.

### 3.8 Neuroactive microbial signatures differ across combinations of VOB and maternal unpredictability

Building on the taxonomic-level analysis that revealed associations of gut microbes and infant VOB, we sought to determine whether the variations in species composition also reflected functional shifts in microbial communities, particularly in relation to neuroactive gene pathways. We hypothesized that microbial gene functions associated with neuroactive metabolites could exhibit enrichment or depletion patterns based on developing levels of VOB and maternal unpredictability due to their correlated topology. Because maternal unpredictability was associated with increased variability in infant VOB responses (Section 3.1), we specifically sought to determine whether this relationship would be distinct at different levels of maternal entropy, which could moderate the emergence of higher levels of VOB associated with microbial genes.

To test whether different sets of genes are enriched or depleted at different levels of phenotypes of interest, we conducted feature set enrichment analysis (FSEA) on microbial neuroactive metabolic pathways using interaction models incorporating infant VOB, maternal unpredictability, and their interaction term (*see Methods 2.16*). We employed logistic regression models to examine the association between gene presence/absence and these behavioral outcomes, while adjusting for control variables (*see Methods 2.13*).

Among the strongest signals, tryptophan synthesis pathways showed strong enrichment associated with infants exhibiting high VOB under elevated maternal unpredictability (ES = +0.226, *q* = 0.020, Figure 4E), while the interaction-associated enrichment score for the same pathway exhibited the opposite directionality (ES = −0.244, *q* = 0.016, Figure 4F). Glutamate synthesis pathways similarly showed enrichment associated with VOB (ES = +0.253, *q* = 0.04, Figure 4E) alongside depletion in the interaction term (ES = −0.214, *q* = 0.134, Figure 4F). At the level of maternal unpredictability (Figure 4D), a number of distinct pathways involved in menaquinone synthesis (ES = −0.153, *q* = 0.102) and GABA synthesis (ES = −0.443, *q* = 0.054) were significantly depleted, while acetate synthesis (ES = −0.138, *q* = 0.120), tryptophan synthesis (ES = +0.152, *q* = 0.056), quinolinic acid degradation (ES = +0.231, *q* = 0.043), and ClpB-associated pathways (ES = +0.303, *q* = 0.112) were enriched.

To further connect these functional shifts to specific microbial taxa, we examined the distribution of neuroactive gene sets across the key genera identified in our taxonomic analyses. Neuroactive gene sets were predominantly carried by commensals of the infant gut that were highlighted in the previous analyses, such as *Bifidobacterium*, *Streptococcus*, *Klebsiella*, and *Escherichia* (Figure 4G). Among these, *Bifidobacterium* genus is an especially predominant carrier of neuroactive gene sets, with highlights to those that were more significantly associated with VOB, such as tryptophan and glutamate synthesis (Figure 4E). Interestingly, GABA synthesis is not carried by certain *Bifidobacterium* spp., being instead split between other genera that were previously implicated in the nonlinear prediction of VOB (Figure 4D). We next examined species-level differences within the key carrier genera. We discovered that, although *B. longum* and *B. breve* are the topmost abundant *Bifidobacterium*, their functional repertoire differs in terms of neuroactive gene sets carried. Notably, *B. longum*, which carries the most abundance of tryptophan synthesis and quinolinic acid degradation, did not contribute at all to glutamate synthesis, which was in turn attributed to *B. breve* and other less prevalent species such as *B. bifidum* (Figure 4H). This finding is consistent with a potential link between microbial composition and neuroactive metabolic plasticity, suggesting that species-level differences in the microbiome are associated with variability in infant behavior in the context of environmental unpredictability, via the metabolic framework enabled by genes carried by different microbial species.

The interaction model revealed that the relationship between microbial neuroactive pathways and infant VOB differed across levels of maternal unpredictability. Specifically, interaction-associated enrichment scores frequently exhibited the opposite directionality from the corresponding VOB-associated effects. This pattern suggests that the positive coupling between elevated VOB and these canonical neuroactive bifidobacterial signatures attenuates under conditions of increased maternal unpredictability, contrary to a simple additive effect. Together, these findings indicate that the distinct microbial neuroactive gene signatures associated with the infant VOB shifts differ systematically across levels of maternal unpredictability.

## 4 Discussion

Our findings support a model in which environmental unpredictability, here measured with maternal behavior, tunes infant behavioral plasticity, measured here in visual orienting shifts response, with microbial community composition shaping the biochemical capacity for infant behavior via neuroactive metabolic pathways. In naturalistic parent-infant interactions, higher maternal unpredictability was associated with more frequent infant visual orienting behavior shifts, a mature-for-age strategy (Figure 2A). Longitudinally, greater maternal unpredictability in early infancy predicted poorer inhibitory control at follow-up. While VOB did not fully explain the relationship between early maternal unpredictability and later inhibitory control scores, higher VOB attenuated the relationship between high maternal unpredictability and later inhibitory control. As shown in Figure 2B, infants exposed to high maternal unpredictability but who exhibited elevated VOB displayed inhibitory control outcomes more comparable to those from low-unpredictability environments with similar VOB, and better than infants in high unpredictability environments but with low VOB.

To summarize, in predictable contexts, a stability-oriented behavioral response (fewer visual orienting shifts) appears as an adaptive, and indeed highly prevalent strategy (~77% of infants showed this pattern), with positive later impact on inhibitory control scores (Figure 2B, blue line). In contrast, in the context of high maternal unpredictability, where the learning signal is ostensibly diminished, infants who shifted gaze frequently, a mature-for-age strategy (Figure 2B, yellow markers), may be attempting to optimize information uptake. This high VOB pattern was observed in a small subset of the sample (41% overall, and ~55% in the context of high maternal unpredictability). In the context of unpredictable caregivers only, this higher VOB strategy was associated with a more resilient later inhibitory control profile (Figure 2B, yellow versus grey lines). We interpret this as a form of phenotypic plasticity. What enables some infants to engage in high VOB for age in the context of maternal unpredictability (*n* = 80, Figure 2A), while a substantial subset (*n* = 65) of infants who experience high maternal unpredictability engaged in a less adaptive but more overall common strategy?

The microbiome converged on a *Bifidobacterium* sp. gradient that tracked VOB in early infancy: higher VOB, which was more than 3x as likely in high maternal unpredictability contexts (Figure 2A, yellow vs. orange markers), aligned with a shift toward *B. breve* and away from *B. longum* (Figure 4). Random-forest rankings reinforced the centrality of *Bifidobacterium* species, with *K. pneumoniae* and *S. salivarius* emerging as secondary signals. Both *B. breve* and *B. longum* are dominant early colonizers that produce neuroactive metabolites including short chain fatty acids (SCFAs), glutamate, and tryptophan-derived products that are implicated in neurotransmission and neurodevelopment (Hensch et al., 1998; Fagiolini and Hensch, 2000; Matsumoto et al., 2013; Janik et al., 2016; Takesian et al., 2018; Ahmed et al., 2022; Fahur Bottino et al., 2025). SCFAs, especially acetate from *Bifidobacterium* sp. (here *B. breve*, Figures 4G–H) regulate microglia and synaptic pruning essential for critical-period plasticity (Erny et al., 2015; Luck et al., 2020). Microbial glutamate production and conversion to GABA modulate E/I balance in developing visual cortex (Hensch et al., 1998; Fagiolini and Hensch, 2000), and microbial tryptophan metabolism yields serotonin and kynurenine-pathway metabolites that stabilize circuits and regulate plasticity (Agus et al., 2018). We interpret our findings in the context of this literature.

Bacterial community composition was not sufficient to explain *adaptive* infant responses (low VOB in low maternal unpredictability contexts and high VOB in high maternal unpredictability contexts). The critical question is what enabled *high* VOB for age in high maternal unpredictability contexts, especially given the overall prevalence of low VOB during dyadic interaction in our age range? Pathway enrichment analyses including the interaction between VOB and unpredictability showed that higher VOB aligned positively with tryptophan-synthesis and glutamate-synthesis enrichments. However, this coupling *attenuated* as maternal unpredictability increased, as reflected by the *opposite* enrichment directionality of the interaction term. One possible interpretation of this pattern is that distinct neuroactive microbial configurations characterize infants exhibiting elevated VOB under conditions of increased environmental challenge.

Consistent with this interpretation, maternal unpredictability-associated signatures additionally included pathways related to GABA synthesis, acetate metabolism and quinolinic acid handling, together suggesting the emergence of an alternative neuroactive metabolic landscape under elevated unpredictability. This broader interaction-associated profile was accompanied by contributions from taxa beyond the dominant bifidobacterial axis observed in the primary VOB associations, including *K. pneumoniae*, *E. coli*, and *S. salivarius*, along with the previously stated contributions from *B. breve* and *B. longum*. Together, these findings are consistent with the possibility that different microbial metabolic configurations may support similar infant attentional phenotypes under different levels of environmental challenge. In this framework, maternal unpredictability is not interpreted as directly modulating the microbiome itself, but instead as an environmental context in which microbiome-associated variation in infant attentional strategies becomes apparent.

Infant-type *Bifidobacterium* sp. cannot fully synthesize tryptophan *de novo* but convert it to indole metabolites (ILA/IAA/I3CA) that activate AhR/Nrf2 and temper inflammation, indirectly affecting tryptophan availability and signaling (Sela et al., 2008; Meng et al., 2020). In more inflamed contexts, host IDO/TDO shunt tryptophan toward kynurenine to quinolinic acid, reducing plasticity-supporting chemistry and increasing stress-linked metabolites (Savitz, 2020), patterns reported under high unpredictability (Hill et al., 2014; Goodwill et al., 2019; Sun et al., 2023). In our data, maternal unpredictability was associated with enrichment of acetate synthesis and enrichment of quinolinic-acid degradation when VOB and the maternal unpredictability by VOB term were included (Figures 4D–F), suggesting a shift under high maternal unpredictability toward an SCFA-supported profile. Acetate, predominant in early infancy and a major *Bifidobacterium* product, can fuel colonocytes, reinforce the barrier, and modulate immune/microglial tone, offering a plausible biochemical scaffold that could help sustain high VOB despite environmental volatility. By contrast, quinolinic acid is a pro-inflammatory, NMDA-agonist kynurenine metabolite; enrichment of its degradation genes is consistent with increased capacity to catabolize/neutralize quinolinic acid under high maternal unpredictability. Together, these findings point to re-routing of tryptophan fate and support in high maternal unpredictability, away from further up-coupling of tryptophan/glutamate and toward acetate-centered support plus quinolinic handling, with species specialization (*B. breve* vs. *B. longum*; Figures 4F,G) and additional contributors (e.g., *K. pneumoniae*, *E. coli*; Figures 4C,F–G) shaping the available pathway mix. As these results reflect gene carriage, confirming pathway activity and causal links will require metabolomic/transcriptomic and inflammatory readouts.

Conceptually, the pattern aligns with developmental plasticity models wherein organisms balance flexibility and stability in response to environmental cues (Frankenhuis and de Weerth, 2013). The findings are also consonant with rodent data linking unpredictable maternal input to accelerated inhibitory maturation (Molet et al., 2016). Here, we implicate microbial tryptophan/indole metabolism as a plausible route from environmental signals to phenotypic plasticity in human infants. In this view, maternal unpredictability is the environmental challenge, VOB is the adaptive strategy, and the microbiome supports or constrains that strategy via neuroactive metabolites. Rather than a one-way path from maternal behavior to microbiome, the data suggest a mediating role for infant behavioral plasticity, with microbial composition reflecting the strategy infants adopt under their environmental conditions. By shaping the biochemical space of neural plasticity, the microbiome may enable resilience that helps infants sustain adaptive function under variable conditions.

Importantly, the present study is observational, and effect sizes for microbiome-behavior associations were modest. Functional inferences are based on gene carriage rather than direct measures of metabolic activity. As such, the findings should be interpreted as identifying associations and generating hypotheses about microbiome-behavior relationships, rather than demonstrating mechanistic or causal pathways. Future studies incorporating longitudinal designs, experimental manipulation, and direct metabolomic or transcriptomic measures will be necessary to establish causal links.

## Data Availability

All data needed to evaluate the conclusions in the paper are present in the paper and/or the Supplementary materials. The raw sequencing data for the Khula study have been deposited in the NCBI Sequence Read Archive (SRA) under BioProject accession number PRJNA1128723. Taxonomic and functional microbial profiles, as well as subject demographics necessary for statistical analyses and machine learning, are available on the Data Dryad under DOI: 10.5061/dryad.15dv41p9k. Information for replicating the package environment and code for data analysis and figure generation, as well as scripts for automated download of input files, are available on GitHub at: https://github.com/Klepac-Ceraj-Lab/PCIVEPMBiome2024 and archived on Zenodo under DOI: 10.5281/zenodo.17707109.
