# Supplementary material for: Microbiome-behavior coupling shapes infant adaptation to early maternal unpredictability
Source: Front Microbiol. 2026 Jun 2;17:1830339. doi: 10.3389/fmicb.2026.1830339 (PMC13269231; doi:10.3389/fmicb.2026.1830339)
Supplement: Supplementary file 1 [file Supplementary_file_1.DOCX]

Supplementary Materials for

**Microbiome-behavior coupling shapes infant adaptation to early maternal unpredictability**

Dima Amso*, Guilherme Fahur Bottino *et al.*

*Corresponding author. Email: da2959@columbia.edu

**This PDF file includes:**

Content page

Supplementary Figure S1 2

Supplementary Table S1 2

Supplementary Note S1 3

Supplementary Figure S2 4

Supplementary Figure S3 5

Supplementary Figure S4 6


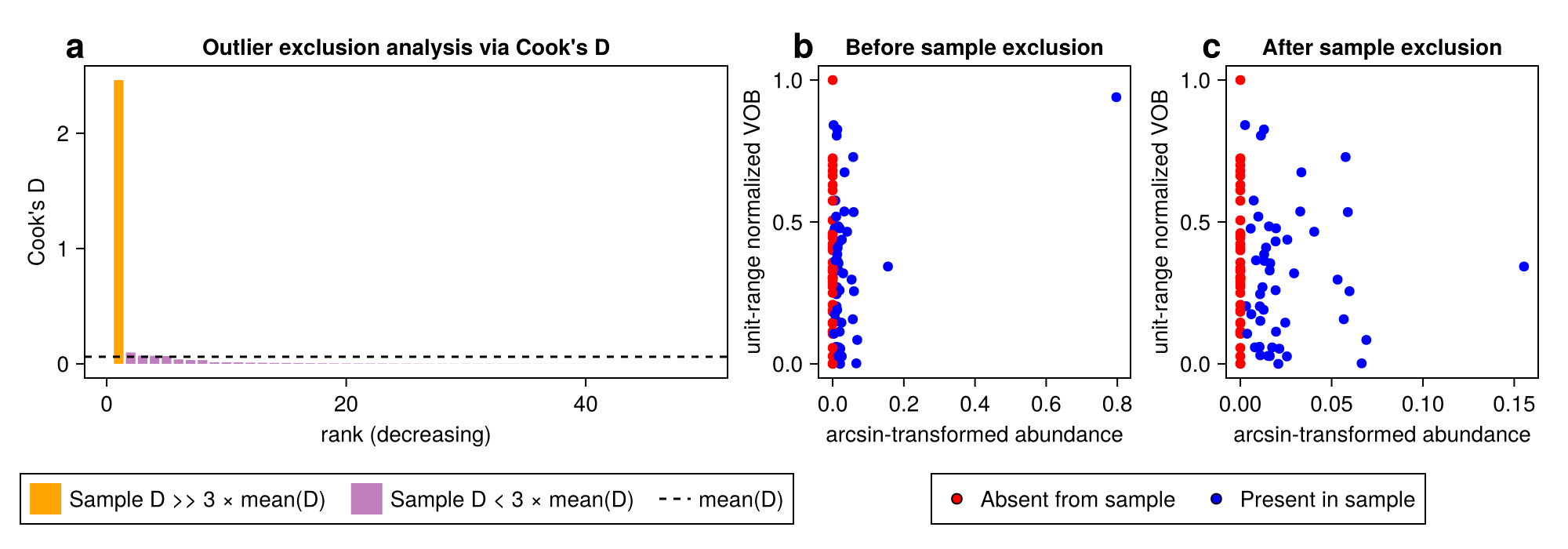


**Figure S1. Sensitivity supplement in support of post-hoc outlier exclusion.** (A) Rank-ordered Cook’s *D* for each sample with nonzero abundance of *Streptococcus mitis*. Dashed line indicates mean D across all samples prior to exclusion. (B, C) unit-range normalized VOB as a function of arcsin-normalized *S. mitis* abundance for before (B) and after (C) exclusion.

**Table S1. Variation in model attributes for top 5 features before and after outlier exclusion.**

| Feature | Coefficient | | p-value | |
| --- | --- | --- | --- | --- |
|  | Before removal | After removal | Before removal | After removal |
| *Bifidobacterium longum* | -0.611 | -0.611 | 0.0004 | 0.0004 |
| ***Streptococcus mitis*** | **0.215** | **-0.011** | **0.0022** | **0.5538** |
| *Bifidobacterium breve* | 0.392 | 0.556 | 0.031 | 0.005 |
| *Klebsiella variicola* | -0.099 | -0.099 | 0.036 | 0.036 |
| *Klebsiella pneumoniae* | -0.131 | -0.159 | 0.046 | 0.031 |

**Supplementary Note S1. Further considerations on control factors and significance framework**

Maternal postnatal depression, as measured by EPDS, was not included in the primary microbiome models. This decision reflects both considerations of data completeness (of which the consequence would be severe loss of power) and the conceptual scope of the analysis. Specifically, EPDS captures maternal affective state transversal to the VOB-unpredictability space, whereas the microbiome analyses are focused on infant-level biological variation associated with behavioral phenotypes. Additionally, the effect of maternal depression in caregiver unpredictability is largely uncharacterized in the demographic of the present study, and this characterization was out of scope for the infant-focused microbial analyses.

The interval between stool collection and PCI (parent-child interaction) assessment was not included as a covariate in primary models. This variable was used exclusively as a filtering criterion to match stool samples to behavioral measurements when multiple visits were necessary to collect data for the same nominal timepoints (~3 months old). Stool collection age and PCI age were highly correlated (R = 0.9); the visits happened less than a week apart for ~65% of participants, and less than 2 weeks apart for ~86% of the participants, minimizing temporal misalignment.

All metagenomic samples were processed using a uniform library preparation protocol and sequenced within a single batch. As a result, sequencing batch and library preparation factors were invariant across samples and were not included as covariates.

The prespecified significance threshold of FDR q < 0.2 applied uniformly across all analyses reflects standard practice in high-dimensional microbiome studies, where the combination of large feature spaces, compositional structure, and correlated tests reduces statistical power under more stringent cutoffs. Accordingly, FDR thresholds in the range of 0.1-0.25 are routinely used in established microbial modeling frameworks such as MaAsLin2^^[[1]](#footnote-1)^^ to maintain sensitivity to distributed, small-to-moderate effect sizes characteristic of microbial community shifts. All reported associations are presented with corresponding effect sizes and exact p-values, enabling full assessment of result strength. In line with this statistical framework, microbiome findings are interpreted as exploratory and hypothesis-generating rather than confirmatory.


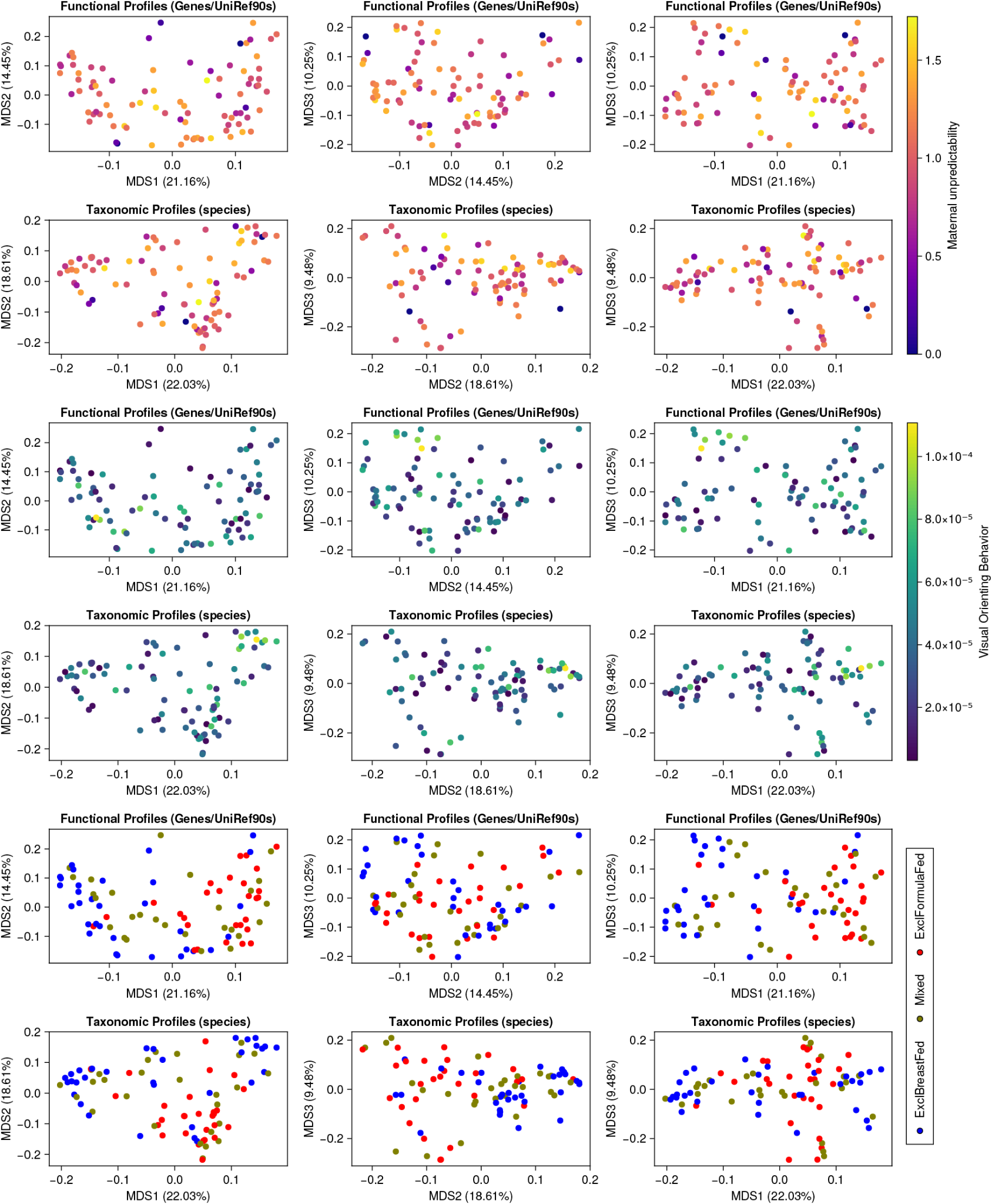
**Figure S2. Principal coordinate analysis (PCoA) by non-metric multidimensional scaling (nMDS) on community-wide beta-diversity calculated as Bray-Curtis dissimilarity.** Plots display all combinations of PC1, PC2 and PC3, for both taxonomic and functional (UniRef90) profiles. Samples (represented by dots) were colored *a posteriori* by associated metadata: maternal unpredictability (A-F), VOB (G-L), and infant feeding practice (M-R).


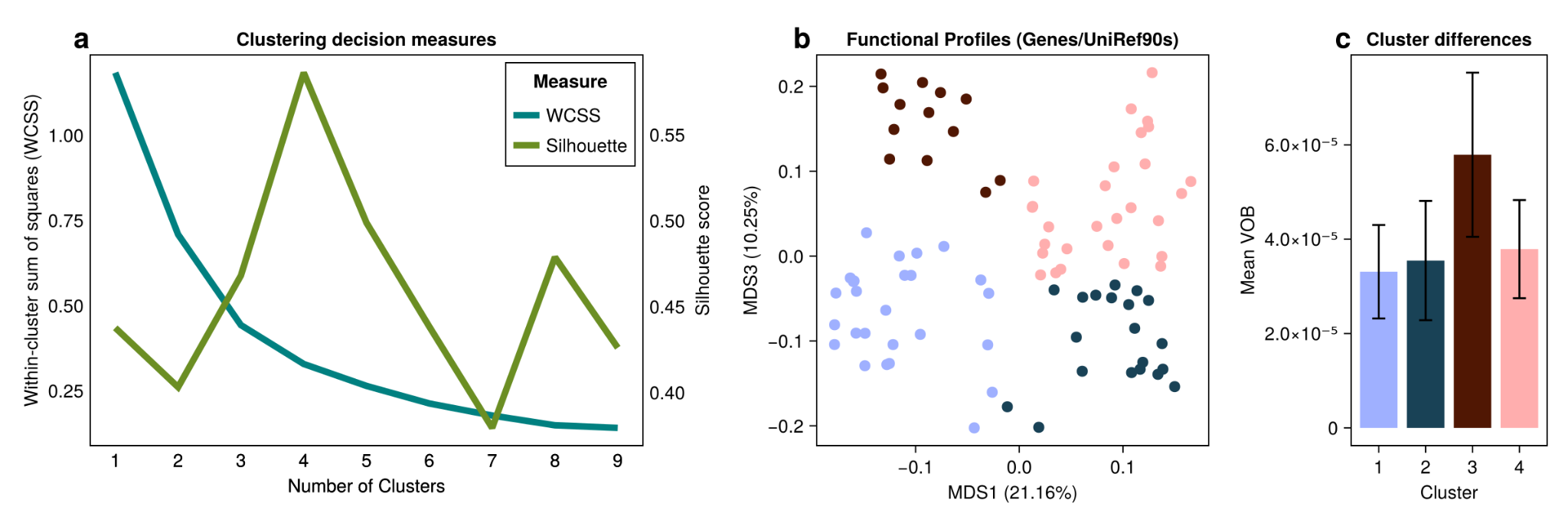


**Figure S3. Cluster analysis of microbiome functional profile ordination.** (A) Within-Cluster Sum of Squares and Silhouette scores for every k = 1:9. K = 4 was chosen as the best number of clusters due to exhibiting the highest silhouette score with a sufficiently low (<50% of k=1) WCSS. (B) Functional profile PCoA **(Main Figure 3b**) colored by k-means clustering assignment with the selected k=4. (C) Mean and SD of Visual Orienting Behavior in each cluster, illustrating the difference between Cluster 4 (salmon) and clusters 1,2,3.


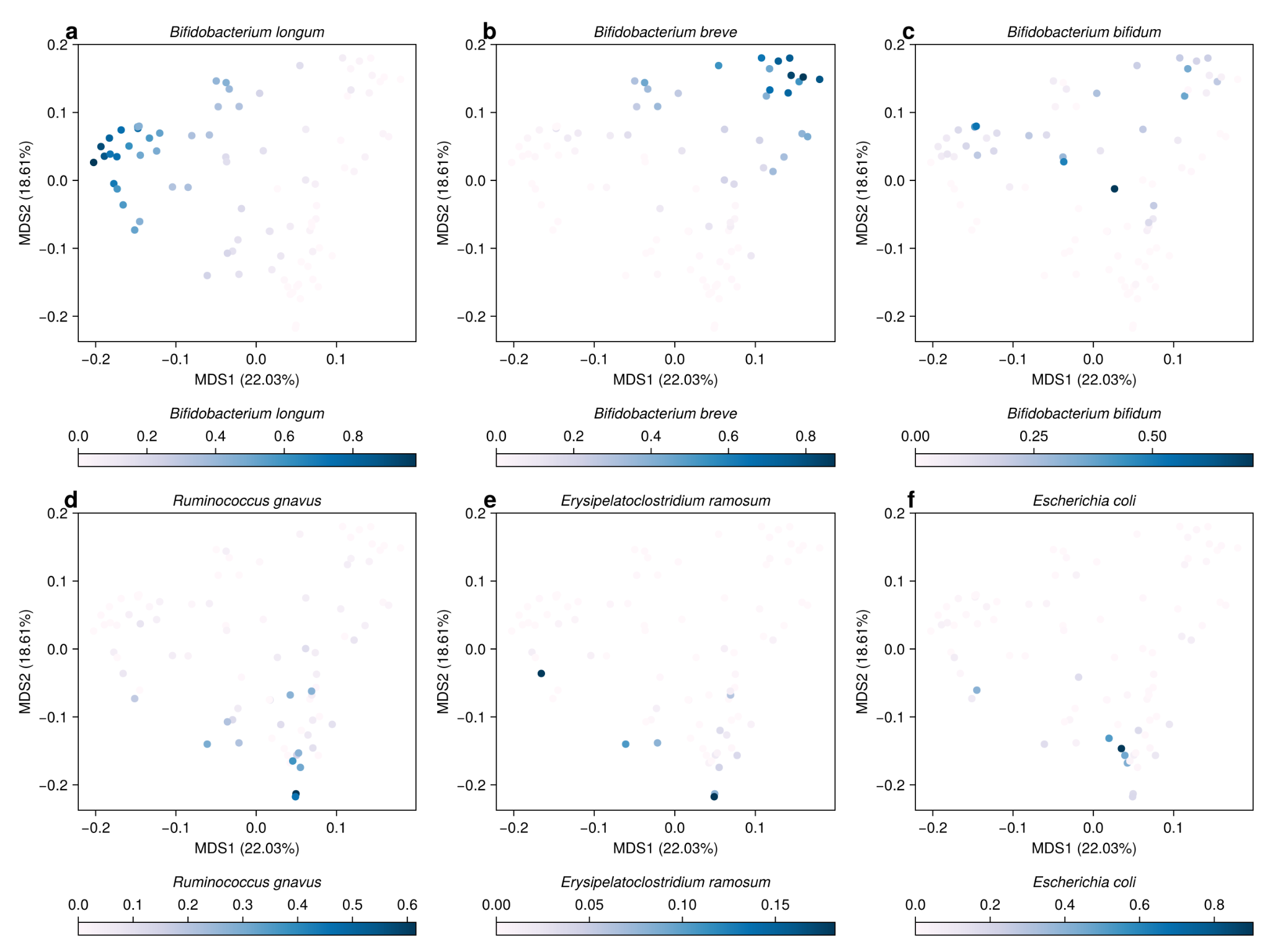


**Figure S4. Select taxa distributed along the principal coordinates of the β-diversity ordination of taxonomic profiles.** Dots are colored by the relative abundance of a particular taxon. Each taxon is listed above its plot. The percent variance explained is indicated on the X and Y axes. The following taxa are plotted: (A) *Bacterium breve*, (B) *Bacterium longum*, (C) *Bifidobacterium bifidum*, (D) *Ruminococcus gnavus,* (E) *Erysipelatoclostridium ramosum*, and (F) *Escherichia coli*.

1. Mallick H, Rahnavard A, McIver LJ, Ma S, Zhang Y, et al. (2021) Multivariable association discovery in population-scale meta-omics studies. PLOS Computational Biology 17(11): e1009442.<https://doi.org/10.1371/journal.pcbi.1009442> [↑](#footnote-ref-1)
